# Social factors and quality of life aspects on frailty syndrome in community-dwelling older adults: the VERISAÚDE study

**DOI:** 10.1186/s12877-018-0757-8

**Published:** 2018-03-07

**Authors:** Carmen de Labra, Ana Maseda, Laura Lorenzo-López, Rocío López-López, Ana Buján, José L. Rodríguez-Villamil, José Carlos Millán-Calenti

**Affiliations:** Universidade da Coruña, Gerontology Research Group, Instituto de Investigación Biomédica de A Coruña (INIBIC), Complexo Hospitalario Universitario de A Coruña (CHUAC), SERGAS, 15071 A Coruña, Spain

**Keywords:** Frailty, Social resources, Quality of life, Elderly

## Abstract

**Background:**

Frailty is a multidimensional clinical geriatric syndrome that may be reversed in its early stages. Most studies have paid attention to its physical or phenotypic boundaries, however, little is known about the social aspects surrounding this geriatric syndrome. The study examined the relationship between socio-demographic factors, social resources, quality of life and frailty in older adults.

**Methods:**

This cross-sectional study included a representative sample (*n* = 749) of adults aged ≥65 years enrolled in forty-three senior centers located in North-West Spain. Socio-demographic data, social resources by the Older Americans Resources and Services Scale, quality of life by the World Health Organization’s Quality of Life measure-brief version (WHOQOL-BREF), and frailty status diagnosed by the Frailty phenotype were measured.

**Results:**

Female gender, age older than 75 years, single marital status, a poor quality of life, and low scores in the physical health domain of the WHOQOL-BREF were the main determinants of being non-robust. Together, these variables explained 24.4% of the variance. Age between 80 and 89 years, and a poor quality of life were the main determinants for non-robust men, whilst the physical health domain of the WHOQOL-BREF was the single main determinant for women.

**Conclusions:**

Our study found evidence that physical frailty is associated with social determinants and several quality of life domains. More research on this understudied topic is needed to avoid healthcare expenditures and improve the quality of life of non-robust elders.

## Background

Frailty is a multidimensional geriatric syndrome widely studied in recent decades. This state of vulnerability to stressors is a result of a decrease or underlying dysregulation of multiple physiological reserves [1]. Different models could be followed when studying frailty. One of them understands frailty as a result of a comprehensive assessment that includes several deficits related with the process of aging, as well as social and psychological aspects [2]. Another approach defines frailty according to a physical phenotype, based on the presence of three or more of the following criteria: unintentional weight loss, self-reported exhaustion, weakness (grip strength), slow walking speed, and low physical activity [1]. Considering the presence or absence of these five identifiable physical alterations, people would be classified as non-frail or robust, pre-frail, and frail. Although Fried phenotype has been the most extensively used instrument to identify frailty [3–5], researchers are also proposing that frailty should shift its focus from an organ or disease based model toward one that is based upon the well-being of the whole person [6].

The prevalence of frailty increases with age [4] and this is important mainly for two reasons. The first one is because frailty exhibits a direct relationship with adverse consequences, such as the risk of falls, disability, hospitalization, and poor survival [1, 2], and the second one is because the world’s population is aging. These two factors will imply, in short, not only a high number of older adults, but also frail elder people with an elevated risk of death. But not only frailty increases with age, there are other circumstances such as severity of functional dependence, and a decrease in social support closed related with the elderly population. Several social factors, such as living alone, feeling of loneliness, access to social resources, or quality of life have been considered as a public health concern and significant risk factors for disability, morbidity and mortality among older people [7–10]. In line with this, a meta-analysis performed with 308.849 subjects, followed for an average of 7.5 years, found a 50% of probability of survival for subjects with adequate social relationships [7].

Research on frailty and its related factors is spreading quite rapidly. Among the factors that may modify the pathways from the occurrence of frailty to adverse consequences, several studies support the importance of psychosocial factors [11–13]. However, and although psychosocial vulnerability has been linked to frailty [14], to our knowledge no studies have explored sufficiently the issue of frailty and isolated social factors and quality of life assessed with a standardized full-scale. Exploring social risk factors and quality of life and their influence on frailty would be very useful to help investigators to develop effective health-related strategies aimed to reduce medical expenses in older adults and plan interventions to prevent risk of mortality, disability or institutionalization.

Bearing this in mind, this study aims to investigate the relationship between socio-demographic, social factors and quality of life with frailty syndrome. Most previous studies involved frail and not pre-frail older adults, where the preventive interventions should be addressed. Thus, we developed our investigation in a large sample of robust (non-frail) and non-robust (pre-frail and frail) community-dwelling older adults.

## Methods

### Participants and procedure

The data for this study derive from the VERISAÚDE “Effectiveness of the Comprehensive Gerontological Assessment (CGA) and longitudinal follow-up in the healthy aging promotion” project. This is a cross-sectional population-based study of community-dwelling individuals aged 65 years and over with a representative sample (*n* = 749), residing in a North-Western region of Spain (Galicia). A more detailed description of the setting, sample selection and data collection has been published previously [15, 16].

To be eligible to participate in the project, participants had to meet the following inclusion criteria: age ≥ 65 years, being actively enrolled in an association or senior center; and ability to read and sign the informed consent form. Exclusion criteria included any participant unable to participate in the gerontological assessment.

Ethical approval was given by the Ethics Committee of the University of A Coruña and was in conformity with the principles embodied in the Declaration of Helsinki. Research participants signed informed consent form before enrollment.

The manuscript was written according to the STrengthening the Reporting of OBservational studies in Epidemiology (STROBE) statement [17, 18].

### Variables and measures

#### A. Sociodemographic aspects and social resources

Age, sex and level of education (≤ 8 years elementary school, 9–17 years and vocational education, > 17 years of college and university) were collected.

We measured social support by items from the Spanish version [19] of the Older Americans Resources and Services (OARS) standardized questionnaire [20]. We assessed the total resources by the nine items total raw score, coded into six categories: (a) excellent, (b) good, (c) mild impairment, (d) moderate impairment, (e) severe impairment, and (f) total impairment. Besides, other items from the OARS scale were also assessed independently: Marital status (single, married, widowed, divorced or separated); Who lives with the person (the item used was: “Who lives with you?”), and Frequency of feelings of loneliness (the item used was: “Do you find yourself feeling lonely quite often, sometimes, or almost never?”).

#### B. Quality of life

The Spanish adaptation [21] of the World Health Organization’s Quality of Life measure-brief version (WHOQOL-BREF) was used to assess the quality of life (QoL) [22]. This instrument, derived from the WHOQOL-100, comprises 26 items. Two of the items are from the overall quality of life and general health facet, and the rest of the 24 items are from the satisfaction section that it is divided into four domains [23]: physical health (containing 7 items-questions Q3, Q4, Q10, Q15, Q16, Q17 and Q18), psychological (containing 6 items- Q5, Q6, Q7, Q11, Q19 and Q26), social relationships (containing 3 items- Q20, Q21 and Q22) and environment (containing 8 items- Q8, Q9, Q12, Q13, Q14, Q23, Q24 and Q25). Each item of the questionnaire is scored from 1 to 5. The mean score of items within each domain is used to calculate the domain score. Mean scores are then multiplied by 4 in order to make domain scores comparable with the scores used in the WHOQOL-100, with higher scores denoting higher self-rated QoL.

#### C. Frailty

The frailty status of each participant was determined according to the five physical criteria proposed by Fried et al. [1]. In brief, these criteria include (i) unintentional weight loss (i.e. not due to dieting or exercise): at least 4.5 kg in past year. (ii) Self-reported exhaustion: identified by two questions from the modified 10-item Center for Epidemiological Studies-Depression (CES-D) scale in its Spanish version [24]. (iii) Weakness: grip strength in the lowest 20% at baseline, adjusted for sex and body mass index. (iv) Slow walking speed: the slowest 20% at baseline, based on time to walk 4.57 m, adjusting for sex and standing height. (v) Low physical activity: the lowest 20% at baseline, based on a weighted score of kilocalories expended per week, calculated according to the Spanish validation of the Minnesota Leisure Time Activity (MLTA) questionnaire [25], according to each participant’s report, and adjusting for sex. Participants with ≥3 positive criteria were defined as frail, with 1–2 positive criteria as pre-frail, and participants without positive criteria as non-frail. Due to the limited number of people classified as frail, two comparison groups were established in the present study: robust (non-frail participants) versus non-robust (frail and pre-frail participants).

### Statistical analysis

Descriptive statistics were used to describe the sample. Quantitative variables were shown as mean and standard deviation and qualitative variables as absolute values and percentage. Parametric tests are applied in this study due to the large sample size. Comparisons between variables were made using Pearson’s chi-square tests for qualitative variables and Student t-tests for quantitative variables. The z test for the comparison of column proportions was used. To determine the clinical significance of differences, effect sizes were estimated in terms of Cohen’s *d* [26] to identify differences in group mean values. “Small ES” (*d* = 0.2), “medium ES” (*d* = 0.5) and “large ES” (*d* = 0.8) benchmarks were used to interpret the ES magnitude. For difference among proportions Cohen’s *h* [26] was calculated, considering “small ES” (*h* = 0.2), “medium ES” (*h* = 0.5) and “large ES” (*h* = 0.8) benchmarks.

The correlations between the dependent variable (frailty score) and the rest of the variables were performed using Pearson’s correlation coefficient in quantitative variables and Spearman’s test for ordinal variables. Cohen’s *q* value [26] was calculated for comparing correlations, using the benchmarks for ‘small ES’ (*q* = 0.10), ‘medium ES’ (*q* = 0.30) and ‘large ES’ (*q* = 0.50).

In order to determine which variables modified a dichotomic dependent variable, a multiple stepwise logistic regression analysis was made using that dichotomic variable as the dependent variable and the other variables (sociodemographic aspects and social resources, and quality of life- two general items and 4 domains) introduced in the model as co-variables. Categorical variables were converted in dichotomous dummy variables for inclusion in the multivariate models. 95% confidence interval (CI) and odds ratio (OR) were calculated for each covariate included in the model. The level of significance was defined as *p* < 0.01. The software package IBM SPSS Statistics v.23.0 (Armonk, NY: IBM Corp., USA) was used to perform all statistical analyses. 

## Results

Descriptive characteristics of the sample are shown in Table 1. The mean age of the participants was 75.8 (SD 7.2) years, being 295 (39.4%) men and 454 (60.6%) women. From them, 105 men and 78 women were considered robust (24.4%), and 190 men and 376 women were considered non-robust (75.6%). 60.2% of our sample had the lowest educational level, and the majority of the sample were married or widowed (88.6%). Among the sociodemographic variables, all of them showed a significant association with the frailty status (age, *p* < 0.0001; gender, *p* < 0.0001; educational level, *p* = 0.047, and marital status, *p* < 0.0001). Z test of proportions revealed statistical differences among variables, being less robust those female older adults with ≤8 years of schooling and those who were widowed. In contrast, being married was statistically associated with being robust. Regarding social support, we found a higher and significant proportion of non-robust people living alone (*p* = 0.002), with their spouse (*p* < 0.0001), or parents (*p* = 0.009). For the WHOQOL-BREF, the satisfaction with the health (*p* < 0.010) and the physical health (*p* < 0.0001) and psychological (*p* = 0.005) domains were associated with being non-robust.

**Table 1:** Characteristics of the sample according to frailty status, robust versus non-robust (pre-frail and frail state)

	Total (*n* = 749)		Robust (*n* = 183)		Non-robust (*n* = 566)		*p*-value	Effect size (Cohen’s *h/d*)
Age (years), mean (SD)^b^	75.8	(SD 7.2)	72.3	(SD 5.1)	76.8	(SD 7.3)	< 0.0001**	*d* = − 0.659
Sex, n (%)^a^							< 0.0001**	
Men	295	(39.4%)	105	(57.4%)	190	(33.6%)		*h* = 0.466
Women	454	(60.6%)	78	(42.6%)	376	(66.4%)		*h* = − 0.467
Education, years, n (%)^a^							0.047*	
≤ 8	451	(60.2%)	96	(52.5%)	355	(62.7%)		*h* = −0.203
9–17	179	(23.9%)	53	(29.0%)	126	(22.3%)		*h* = 0.161
≥ 18	119	(15.9%)	34	(18.6%)	85	(15.0%)		*h* = 0.107
Marital status, n (%)^a^							< 0.0001**	
Single	56	(7.5%)	17	(9.3%)	39	(6.9%)		*h* = 0.073
Married	433	(57.9%)	129	(70.5%)	304	(53.8%)		*h* = 0.353
Widowed	230	(30.7%)	31	(16.9%)	199	(35.2%)		*h* = −0.416
Divorced or separated	29	3.9%)	6	(3.3%)	23	(4.1%)		*h* = −0.055
Who lives with you…?, n (%)^a^								
No one	193	(25.8%)	31	(16.9%)	162	(28.7%)	0.002**	*h* = −0.287
Spouse	429	(57.4%)	127	(69.4%)	302	(53.5%)	< 0.0001**	*h* = 0.310
Children	216	(28.9%)	58	(31.7%)	158	(28.0%)	0.333	*h* = 0.088
Grandchildren	57	(7.6%)	15	(8.2%)	42	(7.4%)	0.735	*h* = 0.038
Parents	15	(2.0%)	8	(4.4%)	7	(1.2%)	0.009**	*h* = 0.203
Brothers and sisters	24	(3.2%)	6	(3.3%)	18	(3.2%)	0.951	*h* = 0.000
Other relatives	52	(7.0%)	15	(8.2%)	37	(6.5%)	0.446	*h* = 0.038
Non-related paid helper	3	(0.4%)	0	(0.0%)	3	(0.5%)	0.323	*h* = −0.451
Others	9	(1.2%)	0	(0.0%)	9	(1.6%)	0.086	*h* = − 0.284
Frequency of feelings of loneliness, n (%)^a^							0.132	
Quite often	43	(5.8%)	6	(3.3%)	37	(6.6%)		*h* = −0.188
Sometimes	127	(17.0%)	27	(14.8%)	100	(17.8%)		*h* = −0.081
Almost never	576	(77.2%)	150	(82.0%)	426	(75.7%)		*h* = 0.147
Social Resources Rating, n (%)^a^							0.448	
Excellent	239	(32.7%)	66	(36.5%)	173	(31.5%)		*h* = 0.105
Good	321	(43.9%)	82	(45.3%)	239	(43.5%)		*h* = 0.020
Mild Impairment	103	(14.1%)	21	(11.6%)	82	(14.9%)		*h* = −0.795
Moderate Impairment	35	(4.8%)	7	(3.9%)	28	(5.1%)		*h* = 0.256
Severe Impairment	21	(2.9%)	4	(2.2%)	17	(3.1%)		*h* = −0.064
Total Impairment	12	(1.6%)	1	(0.6%)	11	(2.0%)		*h* = − 0.084
How would you rate your quality of life?, n (%)^a^							0.069	
Very poor	3	(0.4%)	0	(0.0%)	3	(0.5%)		*h* = −0.200
Poor	15	(2.0%)	1	(0.5%)	14	(2.5%)		*h* = −0.148
Neither poor nor good	223	(29.8%)	47	(25.7%)	176	(31.1%)		*h* = − 0.111
Good	386	(51.5%)	96	(52.5%)	290	(51.2%)		*h* = 0.040
Very good	122	(16.3%)	39	(21.3%)	83	(14.7%)		*h* = 0.157
How satisfied are you with your health?, n (%)^a^							0.010*	
Very dissatisfied	9	(1.2%)	1	(0.5%)	8	(1.4%)		*h* = 0.000
Dissatisfied	28	(3.7%)	1	(0.5%)	27	(4.8%)		*h* = − 0.251
Neither satisfied nor dissatisfied	134	(17.9%)	24	(13.1%)	110	(19.4%)		*h* = −0.164
Satisfied	424	(56.6%)	112	(61.2%)	312	(55.1%)		*h* = 0.122
Very satisfied	154	(20.6%)	45	(24.6%)	109	(19.3%)		*h* = 0.145
Physical health- WHOQOL-BREF score, mean (SD)^b^	14.1	(SD 2.1)	14.9	(SD 1.9)	13.8	(SD 2.1)	< 0.0001**	*d* = 0.536
Psychological WHOQOL-BREF score, mean (SD)^b^	14.3	(SD 1.9)	14.7	(SD 1.8)	14.2	(SD 2.0)	0.005**	*d* = 0.256
Social relationships WHOQOL-BREF score, mean (SD)^b^	13.9	(SD 2.5)	14.1	(SD 2.5)	13.8	(SD 2.5)	0.207	*d* = 0.120
Environment WHOQOL-BREF score, mean (SD)^b^	13.7	(SD 1.8)	13.7	(SD 1.9)	13.6	(SD 1.8)	0.563	*d* = 0.055

*WHOQOL-BREF* World Health Organization Quality of Life- Brief Form; ^a^Chi-squared test. ^b^*t*-test; *Significant (*p*-value) < 0.05; **Significant (*p*-value) < 0.01

The correlations between frailty and socio-demographic characteristics, social resources factors, and quality of life were calculated for the total population and distributed by gender (see Table 2). For the total population, education, quality of life, satisfaction with the health, and three out of four domains of the WHOQOL-BREF: physical health, psychological and social relationships, showed a significant negative correlation with frailty score. A significant positive correlation with age and social resources was also found. In men, a positive correlation with age, and negative correlation with quality of life, and the same three domains of the WHOQOL-BREF, this is, physical health, psychological and social relationships, was observed. In women, the results were similar than in the total sample except in the social relationships domain of the WHOQOL-BREF that was not significant. In terms of difference between amounts of relationship according to gender, small Cohen’s *q* effect sizes were observed in the significant correlation values.

**Table 2:** Correlations between frailty and socio-demographic characteristics, social resources factors, and quality of life

	Frailty			
	Total	Men	Women	Effect size (Cohen’s *q*)
Age (Years)^a^	0.326**	0.314**	0.319**	*q* = − 0.006
Education, years^a^	− 0.100**	− 0.100	− 0.095*	*q* = − 0.005
Social Resources Rating^b^	0.108**	0.062	0.102*	*q* = −0.040
How would you rate your quality of life?^b^	−0.128**	−0.124*	− 0.120*	*q* = − 0.004
How satisfied are you with your health?^b^	−0.211**	− 0.097	−0.265**	*q* = 0.174
Physical health- WHOQOL-BREF score^a^	− 0.352**	−0.206**	− 0.389**	*q* = 0.202
Psychological WHOQOL-BREF score^a^	− 0.176**	−0.147*	− 0.159**	*q* = 0.012
Social relationships WHOQOL-BREF score^a^	− 0.085*	−0.127*	− 0.092	*q* = − 0.035
Environment WHOQOL-BREF score^a^	− 0.050	−0.012	− 0.047	*q* = 0.035

^a^Pearson’s correlation coefficient; ^b^Spearman’s correlation coefficient; *WHOQOL-BREF* World Health Organization Quality of Life- Brief Form; *Significant (*p*-value) < 0.05; **Significant (*p*-value) < 0.01

All the variables were entered into a forward stepwise logistic regression analysis to determine which variables modified the dichotomic dependent variable (frailty status- robust vs. non-robust) (see Table 3, being included only those significant variables). No multicollinearity was observed between the general items and the 4 domains of the WHOQOL-BREF. The values for Variance Inflation Factor (VIF) for the independent variables were between 1.214 and 1.402 in the total sample, between 1.209 and 1.370 in men and between 1.190 and 1.496 in women. Tolerance values were between 0.713 and 0.823 in the total sample, between 0.730 and 0.827 in men and between 0.669 and 0.840 in women. Values of VIF lower than 10 or values of tolerance larger than 0.1 imply no multicollinearity diagnostic [27, 28]. For the total population, the main determinants of being non-robust were being female, being older than 75 years, being single, having rated a poor quality of life, and obtaining low scores in the physical health domain of the WHOQOL-BREF. The combined effects of these predictors simultaneously explain the 24.4% of being a non-robust person. The regression analysis identified different risk factors, depending on the gender. For men, the main determinants of being non-robust were being between 80 and 89 years and having rated a poor quality of life. The combination of having these factors places the risk of being non-robust in a 17.5% in men. For women, the single main determinant was the physical health domain, reaching a prediction of 9.4% of being non-robust.

**Table 3:** Multiple stepwise logistic regression of major social determinants and frailty status

	Total			Men			Women		
	Total B	*p-*value	Odds ratio (95% CI)	Total B	*p-*value	Odds ratio (95% CI)	Total B	*p-*value	Odds ratio (95% CI)
Gender (Female)	−0.968	< 0.0001**	0.380 (0.262–0.552)						
Age (75–79 years)	−0.560	0.012*	0.571 (0.370–0.883)						
Age (80–84 years)	−1.344	< 0.0001**	0.261 (0.146–0.467)	−1.613	< 0.0001**	0.199 (0.085–0.468)			
Age (85–89 years)	−3.837	< 0.0001**	0.022 (0.003–0.158)	− 3.106	0.003**	0.045 (0.006–0.337)			
Age (> 90 years)	−2.697	0.009**	0.067 (0.009–0.517						
Marital status (Single)	0.691	0.044*	1.996 (1.020–3.904)						
How would you rate your quality of life? (Poor-Good)	−0.633	0.014*	1.883 (1.136–3.119)	0.872	0.012*	2.391 (1.212–4.718)			
Physical health- WHOQOL-BREF (score < 14.2857)	0.685	0.001**	1.983 (1.343–2.929)				1.333	< 0.0001*	3.793 (2.222–6.473)
% Correctly predicted (cut-off value of 0.5)			24.4			17.5			9.4

*B* regression coefficient B, *CI* confidence interval; *Significant (*p*-value) < 0.05; ** Significant (*p*-value) < 0.01

## Discussion

In this prospective study, involving 749 community-dwelling individuals, the relationship between frailty and sociodemographic factors, social resources and quality of life aspects were examined. The prevalence of being non-robust in our population (75.6%) is higher than most of the estimates of the prevalence in elderly individuals living in the community (frailty and pre-frailty), which is placed between 46.7% and 78.2%, with approximately 50% as the most common value [29]. These differences can be explained because the different operationalizations used to define a non-robust person. If we separately consider individuals with the intermediate pre-frailty and frailty status, our results identified most participants as pre-frail (71.8%) being only a 3.74% of the people considered as frail (see [30]). The low percentage of frail people could be due to the specific characteristics of our sample, being community-dwelling subjects, living at their home and attending to senior centers.

According to the results of the logistic regression analysis and for the total sample, we found that the predictors of being non-robust spanned different socio-demographic and QoL factors. To be more specific, non-robust frailty criteria were associated with being female, being older than 75 years, being single, having a poor quality of life, and the physical health domain of the WHOQOL-BREF. It is well established in the literature that female gender and being older are two main characteristics related with frailty [1, 4, 31, 32]. Women have a higher likelihood of frailty given that they have a longer life expectancy and accumulate more frailty characteristics and besides, they have lower levels of body mass and muscle strength than the men [4]. Our findings support this conclusion, adding that being single is also a predictor of frailty. The main domains of frailty affected in unmarried people are unintentional weight loss, daily energy expenditure, and exhaustion, explained by a lack of psychological support and economic benefits linked to marriage and the social structure of some older populations, in which, as in our sample, food shopping and preparation is usually done by women [33]. No significant association was observed between perceived social support and frailty status in the current study. A similar lack of relationship was previously described [34]. In contrast, a poor psychosocial adjustment (considering social isolation and feelings of loneliness) associated with the increase of physical frailty status was found [35]. Considering the broad concept of QoL, in this study, we analyzed the general facets and the satisfaction with the health on overall QoL, and the four domains of the WHOQOL-BREF: physical health, psychological, social relationships and the environment. It is well established that as people age the medical conditions increases. Although it is important to treat these medical conditions in order to score more on physical health domain, the absence of them does not necessarily guarantee to have a good, or even adequate QoL. Functional impairment, psychological and social problems could also have a negative impact on QoL [36]. Recently, it has been published a revision showing evidence of a link between QoL and frailty in community-dwelling older people [37]. Specifically, this study reviewed 11 cross-sectional studies, in which worse frailty status was consistently associated with lower levels of QoL, independently of the instruments used to measure these variables. Using the Fried Frailty Index (FFI) and the WHOOQOL-BREF, our results agree with the results of this review, revealing a link between non-robust subjects and poor QoL and the physical dimension of the WHOOQOL-BREF. This result, although not surprising as the FFI definition focused on the physical problems that affect older adults, is novel since frailty has been studied in relation to health outcomes, but not until recently in relation to quality of life. Regarding possible gender differences, we found a link between being non-robust men and poor QoL, and between being non-robust women and the physical dimension of the WHOOQOL-BREF. Taking into account the QoL, the consequences of being frail may substantially differ between being men and women, however, the literature on this topic is scarce. In a study, using the SF-12 [38] being frail was associated, among other variables, with health-related quality of life scores, both in men and in women [39].

### Strengths and limitations

The main strength of this investigation is to use the WHOOQOL-BREF test to measure QoL, as it is specifically designed to be administered in elderly people and measures different domains of QoL. Knowing the state of the different domains involved in the quality of life may help to develop specific interventions that could be addressed to maintain QoL at high levels even though subjects fails to maintain a correct level in any other single domain [40, 41]. Another important strength of this investigation is to take into consideration multiple factors (socio-demographic, social support, and quality of life) as potential determinants of frailty in a large representative sample of people living at their home and attending to senior centers. The main limitation of this study is its cross-sectional nature, that it does not allow establishing causal relationships between frailty and all the described variables. Besides, the selection of the sample, that coming from senior centers, could reduce the prevalence rates of frailty or pre-frailty, being only a 3.74% of this sample considered as frail.

## Conclusions

This paper address frailty considering not only the phenotypic boundaries but also the more uncertain social and quality of life domains. We found that female gender, higher age, poor satisfaction with the general facet on overall QoL, and in the physical domain of QoL are determinants of being non-robust. Identifying these modifiable risk factors associated with robustness might contribute to the public health planning in the development of adequate measures to prevent or event revert this condition. Considering all the above findings, we suggest that further work on this topic should address these understudied elements as a mean of saving healthcare expenditures as well as in improving the elder’s autonomy, improving their QoL.

## Abbreviations

CES-D: Center for Epidemiological Studies-Depression
CGA: Comprehensive Gerontological Assessment
CI: Confidence interval
ES: Effect size
MLTA: Minnesota Leisure Time Activity
OARS: Older Americans Resources and Services
OR: Odds ratios
QoL: Quality of life
STROBE: STrengthening the Reporting of OBservational studies in Epidemiology
VIF: Variance inflation factor
WHOQOL-BREF: World Health Organization’s Quality of Life measure-brief version

## References

[1] Fried LP, Tangen CM, Walston J, Newman AB, Hirsch C, Gottdiener J (2001). Frailty in older adults: evidence for a phenotype. J Gerontol A-Biol..

[2] Rockwood K, Howlett SE, MacKnight C, Beattie BL, Bergman H, Hebert R (2004). Prevalence, attributes and outcomes of fitness and frailty in community dwelling older adults: report from the Canadian study of health and aging. J Gerontol A-Biol.

[3] Cesari M, Prince M, Thiyagarajan JA, Araujo De Carvalho I, Bernabei R, Chan P (2016). Frailty: an emerging public health priority. J Am Med Dir Assoc.

[4] Collard RM, Boter H, Schoevers RA, Oude Voshaar RC (2012). Prevalence of frailty in community-dwelling older persons: a systematic review. J Am Geriatr Soc.

[5] Curcio CL, Henao GM, Gomez F (2014). Frailty among rural elderly adults. BMC Geriatr.

[6] Bergman H, Ferrucci L, Guralnik J, Hogan DB, Hummel S, Karunananthan S (2007). Frailty: an emerging research and clinical paradigm—issues and controversies. J Gerontol A Biol Sci Med Sci.

[7] Holt-Lunstad J, Smith TB, Layton JB (2010). Social relationships and mortality risk: a meta-analytic review. PLoS Med.

[8] Lee IC, Chiu YH, Lee CY (2016). Exploration of the importance of geriatric frailty on health-related quality of life. Psychogeriatrics.

[9] Tabue Teguo M, Simo-Tabue N, Stoykova R, Meillon C, Cogne M, Amiéva H (2016). Feelings of loneliness and living alone as predictors of mortality in the elderly: the PAQUID study. Psychosom Med.

[10] Virues-Ortega J, Vega S, Seijo-Martinez M, Saz P, Rodriguez F, Rodriguez-Laso A (2017). A protective personal factor against disability and dependence in the elderly: an ordinal regression analysis with nine geographically-defined samples from Spain. BMC Geriatr.

[11] Dent E, Hoogendijk EO (2014). Psychosocial factors modify the association of frailty with adverse outcomes: a prospective study of hospitalised older people. BMC Geriatr.

[12] Etman A, Kamphuis CB, van der Cammen TJ, Burdorf A, van Lenthe FJ (2015). Do lifestyle, health and social participation mediate educational inequalities in frailty worsening?. Eur J Pub Health.

[13] Hoogendijk EO, van Hout HP, van der Horst HE, Frijters DH, Dent E, Deeg DJ (2014). Do psychosocial resources modify the effects of frailty on functional decline and mortality?. J Psychosom Res.

[14] Andrew MK (2015). Frailty and social vulnerability. Interdiscip Top Gerontol Geriatr.

[15] Lorenzo-López L, Millán-Calenti JC, López-López R, Diego-Diez C, Laffon B, Pásaro E, Valdiglesias V, Maseda A (2017). Effects of degree of urbanization and lifetime longest-held occupation on cognitive impairment prevalence in an older Spanish population. Front Psychol.

[16] Maseda A, Diego-Diez C, Lorenzo-López L, López-López R, Regueiro-Folgueira L, Millán-Calenti JC. Quality of life, functional impairment and social factors as determinants of nutritional status in older adults: the VERISAÚDE study. Clin Nutr. 2017; in press 10.1016/j.clnu.2017.04.009 28456537

[17] Vandenbroucke JP, von Elm E, Altman DG, Gøtzsche PC, Mulrow CD, Pocock SJ (2014). Strengthening the reporting of observational studies in epidemiology (STROBE): explanation and elaboration. Int J Surg.

[18] von Elm E, Altman DG, Egger M, Pocock SJ, Gøtzsche PC, Vandenbroucke JP (2014). The strengthening the reporting of observational studies in epidemiology (STROBE) statement: guidelines for reporting observational studies. Int J Surg.

[19] Grau Fibla G, Eiroa Patiño P, Cayuela DA (1996). Spanish version of the OARS multidimensional functional assessment questionnaire: cross-cultural adaptation and validity measurement. Aten Prim.

[20] Fillenbaum GG (1998). Multidimensional functional assessment of older adults: the Duke older Americans resources and services procedures.

[21] Lucas-Carrasco R (1998). Spanish version of the WHOQOL.

[22] The WHOQOL Group (1998). Development of the World Health Organization WHOQOL-BREF quality of life assessment. Psychol Med.

[23] World Health Organization, Programme on Mental Health (1998). WHOQOL user manual.

[24] Ruiz-Grosso P, Loret de Mola C, Vega-Dienstmaier JM, Arevalo JM, Chavez K, Vilela A (2012). Validation of the Spanish Center for Epidemiological Studies Depression and Zung Self-Rating Depression Scales: a comparative validation study. PLoS One.

[25] Ruiz Comellas A, Pera G, Baena Díez JM, Mundet Tudurí X, Alzamora Sas T, Elosua R, et al. Validation of a Spanish short version of the Minnesota leisure time physical activity questionnaire (VREM). Rev Esp Salud Publica. 2012;86(5):495–508. 10.4321/S1135-57272012000500004 23223762

[26] Cohen J (1988). Statistical power analysis for the behavioural sciences.

[27] Gujarati DN (2010). Econometría Básica.

[28] Montgomery DC, Peck EA. Introduction to linear regression analysis. 2nd ed. New York: John Wiley & Sons; 1992.

[29] Santos-Eggimann B, Cuénoud P, Spagnoli J, Junod J (2009). Prevalence of frailty in middle-aged and older community-dwelling Europeans living in 10 countries. J Gerontol A-Biol..

[30] Lorenzo-López L, López-López R, Maseda A, Diego-Díez C, Gómez-Caamaño S, Millán-Calenti JC (2016). Prevalence and clinical characteristics of Prefrailty in elderly adults: differences according to degree of urbanization. J Am Geriatr Soc.

[31] Romero-Ortuno R, Kenny RA (2012). The frailty index in Europeans: association with age and mortality. Age Ageing.

[32] Woo J, Zheng Z, Leung J, Chan P (2015). Prevalence of frailty and contributory factors in three Chinese populations with different socioeconomic and healthcare characteristics. BMC Geriatr.

[33] Trevisan C, Veronese N, Maggi S, Baggio G, De Rui M, Bolzetta F (2016). Marital status and frailty in older people: gender differences in the Progetto Veneto Anziani longitudinal study. J Women's Health.

[34] Amaral FL, Guerra RO, Nascimiento AF, Maciel AC (2013). Social support and the frailty syndrome among elderly residents in the community. Cienc Saude Coletiva.

[35] Mulasso A, Roppolo M, Giannotta F, Rabaglietti E (2016). Associations of frailty and psychosocial factors with autonomy in daily activities: a cross-sectional study in Italian community-dwelling older adults. Clin Interv Aging.

[36] Jakobsson U, Hallberg IR, Westergren A (2004). Overall and health related quality of life among the oldest old in pain. Qual Life Res.

[37] Kojima G, Iliffe S, Jivraj S, Walters K (2016). Association between frailty and quality of life among community-dwelling older people: a systematic review and meta-analysis. J Epidemiol Commun H.

[38] JJr W, Kosinski M, Keller SD (1996). A 12-item short-form health survey: construction of scales and preliminary tests of reliability and validity. Med Care.

[39] Serrano MD, Garrido M, Fuentes RM, Simón MJ, Díaz MJ (2017). The impact of biological frailty syndrome on quality of life of nursing home residents. Appl Nurs Res.

[40] Layte R, Sexton E, Savva G (2013). Quality of life in older age: evidence from an Irish cohort study. J Am Geriatr Soc.

[41] The WHOQOL Group (1995). The World Health Organization quality of life assessment (WHOQOL): position paper from the World Health Organization. Soc Sci Med.

